# Sex and age characteristics of thunderstorm asthma emergency department visits

**DOI:** 10.1016/j.heha.2024.100099

**Published:** 2024-07-17

**Authors:** M. Luke Smith, Richard F. MacLehose, Chris H. Wendt, Jesse D. Berman

**Affiliations:** a Minnesota Population Center, University of Minnesota School of Public Health, Minneapolis, MN 55455, USA; b Division of Epidemiology and Community Health, University of Minnesota School of Public Health, Minneapolis, MN 55455, USA; c Pulmonary Allergy Critical Care, and Sleep Medicine, Minneapolis VA Health Care System, Minneapolis, MN, USA; d Pulmonary Allergy Critical Care, and Sleep Medicine, University of Minnesota, Minneapolis, MN, USA; e Division of Environmental Health Sciences, University of Minnesota School of Public Health, Minneapolis, MN 55455, USA; f Social Science Research Institute, Penn State University, University Park, PA 16802, USA

**Keywords:** Asthma, Thunderstorm asthma, Pollen, Life course

## Abstract

Severe asthma has been shown to occur in the combined presence of high pollen and thunderstorm conditions, also known as ‘thunderstorm asthma.’ First studied as severe epidemic events, recent longitudinal work studied less dramatic but more frequent occurrences. We explore thunderstorm asthma-related emergency department visits in the Minneapolis-St. Paul metropolitan area and evaluated risk differences by sex and age. We define a thunderstorm asthma exposure event as the daily occurrence of 2 or more lightning strikes during high pollen periods, and use daily counts of asthma-related emergency department visits to estimate relative and absolute risk of severe asthma during thunderstorm asthma events for the full population and for sex and age subgroups. The overall population had a 1.06 (95 % CI: 1.02, 1.09) times higher risk of asthma-related ED visits during thunderstorm asthma events compared to days without thunderstorm asthma events. Children under 18 show no higher risk (RR 1.02; 95 % CI: 0.97 1.08), but adults 18–44 years (RR 1.08; 95 % CI: 1.02, 1.13) and 45 and up (RR 1.08; 95 % CI 1.02, 1.15) show higher relative risk. Absolute risk measures show similar patterns to the age and sex results, but age-sex subgroups show more variation in absolute vs relative risk. Our results support an association between ED visits and thunderstorm asthma and provide evidence of varying risks by sex across the life course. These differences in risk have implications for clinical treatment of this allergic type of asthma and for future research into this poorly recognized environmental exposure.

Plain Language Summary: Recent research has highlighted the existence of Thunderstorm asthma events, a phenomenon in which pollen grains rupture in the conditions that occur with a thunderstorm, releasing subpollen particles that are capable of triggering severe asthma in susceptible populations. Where severe asthma is a disease that usually impacts children, we find in this study that asthma ED visits associated with thunderstorm asthma events more frequently impact adults, particularly males 18–44 and females 45 and up.

## 1. Introduction

Asthma is a disease of the respiratory tract characterized by chronic effects coupled with severe acute attacks (Harver and Kotses, 2010). It is a complex multifactorial disease with multiple definitions and risk factors that pose challenges in epidemiological description (Van Wonderen et al., 2010). Symptoms may include shortness of breath, coughing, and wheezing (Harver and Kotses, 2010). Asthma is associated with allergic (Wenzel, 2012) subtypes and non-allergic subtypes linked to individual factors, such as race, genetics, (Ober and Yao, 2011) diet, or obesity, (Guilleminault et al., 2017; Ford, 2005; Peters et al., 2018) and differential impacts associated with socioedemographics, such as ethnicity (Rona, 2000) and poverty (Nunes et al., 2017). Environmental exposures that exacerbate asthma incidence and severity include mold and cockroach allergens, (Gautier and Charpin, 2017) in addition to air pollution and allergy to pollen (Molnár et al., 2021), (P.E. Taylor et al., 2007).

Asthma affects nearly 7.8 % of U.S. residents as of 2017, (CDC 2017) with higher adult prevalence rates (8 %) compared to children under 18 years (7 %). However, while adults have a higher prevalence, children bear a greater burden for severe asthma outcomes. Nationally, emergency department (ED) visits occur less frequently with increasing age, with an annual rate of 881/1000,000 for children under 18, 627/1000,000 for adults ages 18–34, 369/1000,000 for ages 35–64, and 182/1000,000 for ages 65 and older ((CDC) Centers for Disease Control & Prevention 2021). Females generally show higher rates of ED visits (504/1000,000) compared to males (311/1000,000) ((CDC) Centers for Disease Control & Prevention 2021). However, asthma prevalence is higher in males under 18 compared to females under 18[15], and male children also have a higher incidence of asthma emergency department visits (1031/1000,000) compared to females (725/1000,000) ((CDC) Centers for Disease Control & Prevention 2021). Patterns of risk of severe asthma by sex are reversed over the life course, with higher risk for females who are adults compared to adult males ((CDC) Centers for Disease Control & Prevention 2021), (NIH-NHLBI 2007). These varying patterns in prevalence and incidence of asthma by sex over the life course (Dharmage et al., 2019) can have implications for differential treatment by sex and age (Naeem and Silveyra, 2019).

A unique example of extreme allergic-type asthma occurs in a phenomenon commonly called ‘thunderstorm asthma event.’ (Hew et al., 2019; Packe and Ayres, 1985; D’Amato et al., 2016). While pollen particles are thought to be too large to cause severe asthma, sub-pollen particles can form during atmospheric conditions related to thunderstorms (Taylor et al., 2007). It is hypothesized that pollen grains are carried aloft by thunderstorm-associated updrafts, where, in the presence of moisture and changing air pressure, the pollen particles fracture and release large numbers of sub-pollen particles with diameters <1 mm (D’Amato et al., 2016; Miguel et al., Aug. 2006; Taylor et al., 2002). Downdrafts from the storm subsequently force the sub-pollen particles to ground level, where they concentrate and trigger asthmatic responses in sensitive persons (D’Amato et al., 2016). The biological plausibility of sub-pollen particle creation has been demonstrated in laboratory settings in multiple genera of pollen (Miguel et al., Aug. 2006; Grote et al., 2000; Bacsi et al., 2006). Limited research into previously identified extreme epidemic events suggests that populations affected by allergic-type thunderstorm asthma events are more likely to be male and less likely to be a child (Kevat, Sep. 2020).

Thunderstorm asthma events have been documented in approximately 20 extreme instances (Kevat, Sep. 2020). The most well-studied and highly notable occurrences occurred in Melbourne, Australia, in 2016, where emergency department visits and hospitalizations increased by 672 % and 900 %, respectively, during a thunderstorm asthma event (Thien et al., 2018; Packe and Ayres, 1985). Recent work has explored this phenomenon at less severe atmospheric and pollen levels (Smith et al., 2022) and indicates potential underrecognized risk from thunderstorm asthma exposures. Presently, there is limited exploration into the at-risk populations affected by thunderstorm asthma events, although prior research suggests that this unusual environmental phenomenon impacts different age and sex characteristics than is observed in severe asthma. In this study, we explored the variation in risk of asthma ED visits by age (Thien et al., 2018) and sex (Hew et al., 2019) associated with thunderstorm asthma events in a Midwestern urban region. We seek to develop a better understanding of asthma risk associated with thunderstorm asthma events in relative and absolute risk terms for age and sex subgroups and identify who is most vulnerable to this poorly understood phenomenon.

## 2. Methods

Our study area was zip codes completely or partially within 20 miles of the Minneapolis St-Paul International Airport in Minnesota, United States; a metro region of several million people (Supplemental Fig. S1). Using the Minnesota Hospital Association’s Hospital Uniform Billing Claims data accessed through the Health Economics Program at the Minnesota Department of Health, we extracted daily counts of asthma-related ED visits from January 2007 to October 2018. Diagnosis of asthma was based on a first or secondary diagnosis code using ICD 9 codes of 493 or ICD-10 codes of J45 (after 10/1/2015) (Travers et al., 2014). The primary outcome was the daily count of asthma-related emergency department visits aggregated across the study region. Individual-level information was limited with only sex and age-category sub-counts.

Our primary exposure of a ‘thunderstorm asthma event’ was a thunderstorm, defined as two or more lightning strikes in a day, occurring in the presence of high or very high pollen (≥75th percentile of pollen counts) on the same day (Smith et al., 2022). This was recorded as a binary outcome of ‘no thunderstorm asthma event’ or a ‘thunderstorm asthma event.’ While described elsewhere (Smith et al., 2022), daily lightning was measured with the VAISALA lightning detection network (Vaisala 2015; NCDC 2019), which summarizes daily lightning at a 0.1 × 0.1° grid. We used an inverse distance weighting method to calculate a value for all zip codes in the study area and used the value for the zip code centroid for MSP airport as the value for the study. Our choice of 2 or more strikes was based on the National Weather Service’s storm definition (Sampson and Lake, 1997). Pollen counts were measured at a single private site in south Minneapolis in close proximity to the airport and extrapolated as the pollen concentration for the study area. Pollen data is collected and counted by National Allergy Bureau certified pollen counters employed by the Clinical Research Institute of Minneapolis; there are no other certified pollen measurement sites in the state of Minnesota (Clinical Research Institute NAB pollen Counting Site 2021). The 20-mile study radius is based on prior studies that suggest correlation of pollen measures within this distance (Katelaris et al., 2004) and research exploring the role of weather on asthma (Celenza et al., 1996; Villeneuve et al., 2005; Davies et al., 2017). Additionally, in previous work exploring the overall association between thunderstorms in the presence of pollen and asthma, we found no heterogeneity of effect at distances up to 20 miles (Smith et al., 2022). Missingness for pollen data was estimated for up to three consecutive missing days as the average of the two bounding days.

Weather confounders, including maximum daily temperature, maximum daily humidity, daily maximum wind speed, and daily total precipitation (Buckley and Richardson, 2012; Lei et al., 2020; Hu et al., 2020; Michelozzi et al., 2009) were acquired from 16 Automated Surface Observation System or Automated Weather Observation System stations throughout the study area. Air pollution measures of ozone and fine particulate matter (PM_2.5_) (Williams et al., Mar. 2019; Karimi et al., 2020; Stieb et al., 1996; Gent et al., 2009; Byrwa-Hill et al., 2020) were collected from 9 ozone and 17 PM_2.5_ monitor sites near the study area and accessed using the EPA’s Air Quality System (“US Environmental Protection Agency Jun. 26, 2021).

Daily values for weather and pollution were assigned to all zip codes in the study area using nearest neighbor methodology to ensure that any missing days had information from other sites. We then used the value of the zip code at the center of the study area (MSP airport) for all daily exposure (Smith et al., 2022).

### 2.1. Statistical approach

Rates (per million people) were estimated for all-cause asthma emergency department (ED) visits in the study area, along with incidence rates by age (under 18, 18–14, 45+ years), sex (male, female), and age-sex subgroups. Total population in our study area zip codes were derived from US Census data, as well as sex and age subgroup populations, from April 2007 through October 2018 (Ruggles et al., 2018).

Our main model estimated the risk of asthma ED visits associated with exposure to thunderstorm asthma events for the pollen months from April to October from 2007 to 2018. We used a quasipoisson regression model to estimate the risk ratio for the crude association, adjusting for day of week and seasonal asthma variation using a 6-degree cubic spline for time. A fully adjusted model was also fit, including time-varying daily environmental exposures of precipitation, wind, PM_2.5_, ozone, and maximum relative humidity. Results were calculated at lags of 0 through 6 days to estimate associations between asthma ED visits and a thunderstorm asthma event that happened up to six days prior. The fully adjusted model takes the form of equation [1]:

(1) $$\log\left[E\left(y_{t}\right)\right]=B_{0}+B_{1}{\mathrm{event}}_{t}+B_{2}{\mathrm{Tmax}}_{t}+B_{3}{\mathrm{PM}25}_{t}+B_{4}{\mathrm{Wind}}_{t}+B_{5}{O3}_{t}+B_{6}{\mathrm{RH}}_{t}+B_{7}{\mathrm{Precip}}_{t}+B_{8}{\mathrm{dow}}_{t}+{\mathrm{offset}}_{t}+g\left(\mathrm{time}\right)$$

The outcome is daily counts of asthma ED visits, *y* on day *t*. *Event* on day t is the primary exposure of presence/absence of a thunderstorm asthma event, *Tmax* is maximum temperature (°C), *PM25* is mean PM_2.5_ (ug/m^3^), *Wind* is maximum wind speed (miles per hour), *O3* is 8 h ozone maximum (ppb), *RH* is maximum relative humidity, *Precip* is total precipitation (mm), *dow* is a categorical term for day of week, and *g(time)* is a 6-knot spline term to control for seasonal and long-term trends in asthma emergency department visits. Sensitivity analyses explored splines with 5 and 7 knots. We include an offset term as the log of the annual target population for each year.

We repeated the crude and fully adjusted analysis by the following age and sex subgroups: 1) entire population, 2) all male, 3) all female, 4) under age 18, 5) 18–44 years, 5) 45 years and up, 6) male under 18, 7) male 18–44, 8) male 45 and up, 9) female under 18, 10) female 18–44, and 11) female 45 and up. The three age categories were selected as a compromise between population size and sufficient groups to investigate known differences in severe asthma across the life course (CDC 2017) “To test differences across groups, we also fit models with interaction terms and tested pairwise interactions between thunderstorm asthma event and sex, as well as thunderstorm asthma event by age, and tested the thunderstorm asthma event by age interaction models within sex subgroups. In these models, different population offsets are included for each subgroup. We evaluated statistical significance of the interaction term(s) using a likelihood ratio test.”

We calculated risk differences as the marginal difference in daily asthma risk on exposed days vs unexposed days for the whole population, and in subgroups using an interaction term for sex (sex by event), age (age by event), and separate models with an age by event interaction term for males and females separately. We used the postestimation “prediction” package in R to get absolute counts and SE of events in exposed and unexposed to calculate the difference of risk in exposed and unexposed, and we calculated confidence intervals using the standard error of difference assuming unequal variance. These confidence intervals are reported per 100,000 population by subgroup for comparison, calculated using the mean population offset for each subgroup for the study period. Using a Z-test we performed a test for additive interactions by comparing the difference of risk differences of severe asthma for people exposed to thunderstorm asthma events compared to people not exposed to severe asthma events by sex, age, and using the separate analyses by sex to test for risk differences by age within sex groups (Di et al., 2017).

We tested the robustness of our thunderstorm asthma definition by exploring alternative exposures that assess the presence of potential confounding due to high pollen or thunderstorms in isolation, but separate from their joint occurrence as a thunderstorm asthma event. First, we evaluated potential ED visit risk associated with 1) pollen only by considering exposure days being high or very high pollen (>75th percentile) in the absence of lightning and 2) lightning only with exposure days being the occurrence of a storm (2+ lightning strikes) during low pollen conditions (<25th percentile). We further tested a model with high pollen (> 75th percentile) and occurrence of a storm (lightning > 2+ strikes) as main effects with an interaction term and assessed the estimated effect of being exposed to both lightning and pollen in this model. As a final sensitivity analysis, we ran the full model for 0–1 day lags restricted to only data with missing information for (1) no more than two consecutive days and (2) data with no missing days.

### 2.2. Research ethics

This study was deemed exempt by the University of Minnesota Institutional Review Board, with access restricted to a secure virtual environment via the Minnesota Department of Health. Data were prepared using SAS version 9.4 (The SAS Institute 2016) and ArcGIS Desktop 10.6.1. Statistical analyses were conducted in R (version 3.6.0) (R Core Team 2020).

## 3. Results

**Fig. 1.**
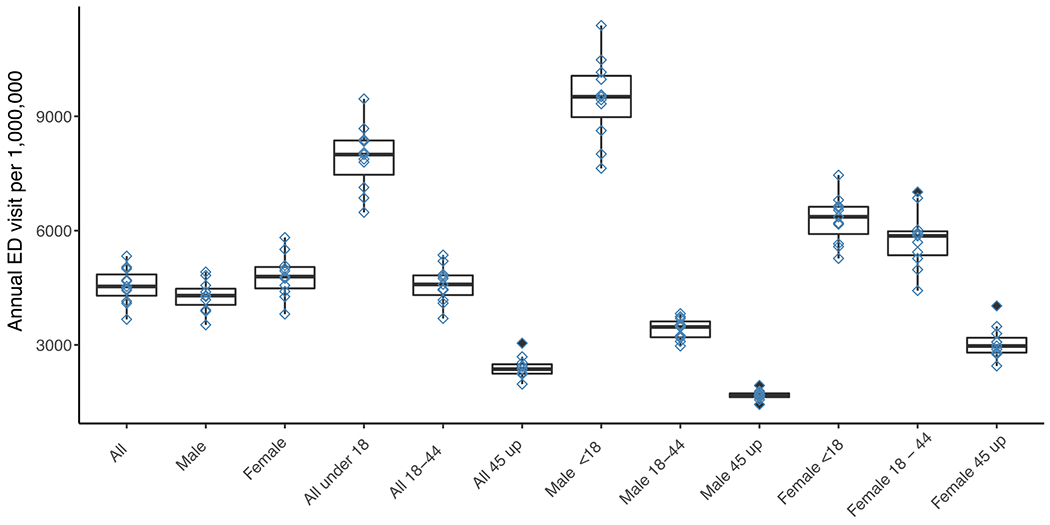
Median and interquartile range of annual rates of severe asthma emergency department visits per million population based on severe asthma admissions in the Minneapolis-St. Paul, Minnesota metropolitan region (2007–2017). Population subgroups are all population, all male, all female, all under 18, 18–44, 45 and up, male under 18, male 18–44, male 45 up, female under 18, female 18–44, female 45 up. Diamonds represent years and solid diamonds are outliers.

To calculate baseline rates of all cause asthma in our study population we first describe cases in years with complete health information. We observed 133,844 total asthma ED visits from April 2007 to October 2017 for an average of 12,168 (972 standard deviation) per year. All-cause rates of asthma ED visits for the total population, by sex, by age, and by age*sex categories are shown in Fig. 1. We estimated an average of 4660 asthma ED visits per million persons per year, with males having slightly lower annual rates than females (4400 vs 4910 per million for males vs. females, respectively). By age group, asthma ED visits showed the highest incidence in young children with median rates decreasing with age. When examining age groups by sex, we observed male children had the highest risk, with 9570 cases per million, and this risk decreased in older age groups with similar but smaller magnitude trends for females.

To estimate the effects of thunderstorm asthma events, we evaluated 142,333 all-cause asthma ED visits from April 2007 to October 1, 2018, the last date of pollen collection in our study area. There were 108 thunderstorm asthma events during the study period of 2441 days. For our alternative exposures, there were 886 days when pollen was high or very high and <2 lightning strikes occurred, and 45 days where two or more lightning strikes occurred, and pollen was below the 25th percentile.

**Fig. 2.**
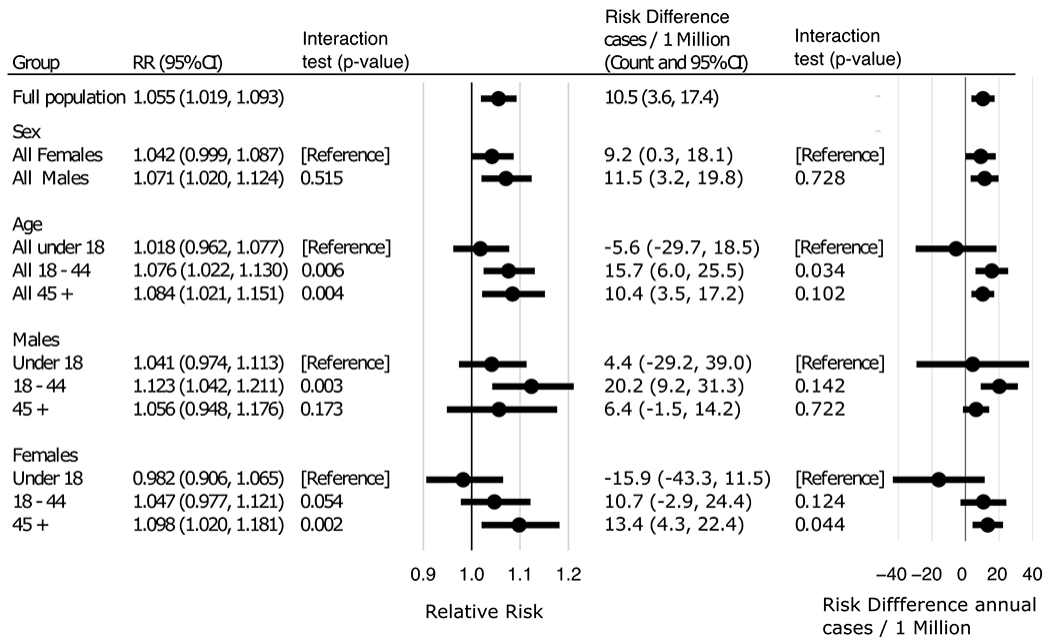
Estimated relative risk and absolute risk differences of asthma-related emergency department visit for thunderstorm asthma by age and sex subgroups. Interaction tests represent p-values for effect differences against the reference group. Interaction term of the absolute cases/million is a Z-test of the difference in estimates with combined standard error.

Adjusted for all covariates, day of week, and seasonal splines, the all-population relative risk of asthma ED visits for people exposed to thunderstorm asthma events vs those not exposed to these events is 1.06 (95 % CI: 1.02, 1.09; Fig. 2). Males showed similar relative risk (RR 1.07; 95 % CI: 1.02,1.12) as females (RR 1.04; 95 % CI: 1.00, 1.09), and in a model with a sex by event interaction there is no evidence associations differ by sex. The relative risk of asthma ED visits associated with thunderstorm asthma events increased with age, with no association for children under 18 years (RR 1.02; 95 % CI: 0.96, 1.08), increasing to a 1.08 times higher risk for adults 18–44 (95 % CI: 1.02, 1.13) and 1.08 times higher risk for adults 45 and up (95 % CI: 1.02, 1.15). Interaction tests of the relative risk revealed some evidence of significantly lower risk for people under 18 compared to those aged 18–44 (*p* = 0.006) and 45 and up (*p* = 0.004).

Stratified by sex, males had the highest relative risk in the 18–44 category (RR= 1.12; 95 % CI: 1.04, 1.21), with lower non-significant relative risk for male children and male adults over 45. Tests of interaction by age in males only shows evidence of interaction when comparing males 18–44 against males under 18 (*p* = 0.003), but not relative to those 45+ years. For females, the relative risk of asthma ED visit following a thunderstorm asthma event increased with age, with a highest relative risk of 1.10 for women over 45 (95 % CI: 1.02, 1.18) and lower RRs for females <18 and females aged 18–44. In a model with an age by event interaction term for females only, we find evidence of difference in relative risk for females 45 up vs. under 18 (*p* = 0.002). Overall, relative risk of asthma ED visit is higher on days with thunderstorm asthma events with greater associations in persons over 18, particularly for males 18–44 and females 45 and up.

Predicted margins were used to examine the absolute difference in asthma ED visits following thunderstorm asthma events compared to non-thunderstorm asthma conditions (Fig. 2). We estimated an additional 10.5 annual ED asthma cases per million persons (95 % CI: 3.6, 17.4) attributed to thunderstorm asthma events. For men, there were 11.5 (95 % CI: 3.2,19.8), and for women, 9.2 (95 % CI: 0.3,18.1) additional cases/million. Looking at age subgroups, people under 18 experienced 5.6 (95 % CI: −29.7, 18.5) fewer cases per million, while people 18–44 and 45 and up experienced 15.7 (95 % CI: 6.0, 25.5) and 10.4 (95 % CI: 3.5, 17.2) additional cases per million, respectively. For males, the 18–44 age group has the highest change in absolute risk of any subgroup with 20.2 (95 % CI: 9.2,31.3) additional cases/million. Males under 18, and over 45 showed little change in absolute risk. For females, under 18 showed a non-significant protective effect with −15.9 cases per million (95 % CI: −43.3, 16.0), while females 45 and up experienced an additional 13.4 cases per million (95 % CI: 4.3, 22.4).

**Fig. 3.**
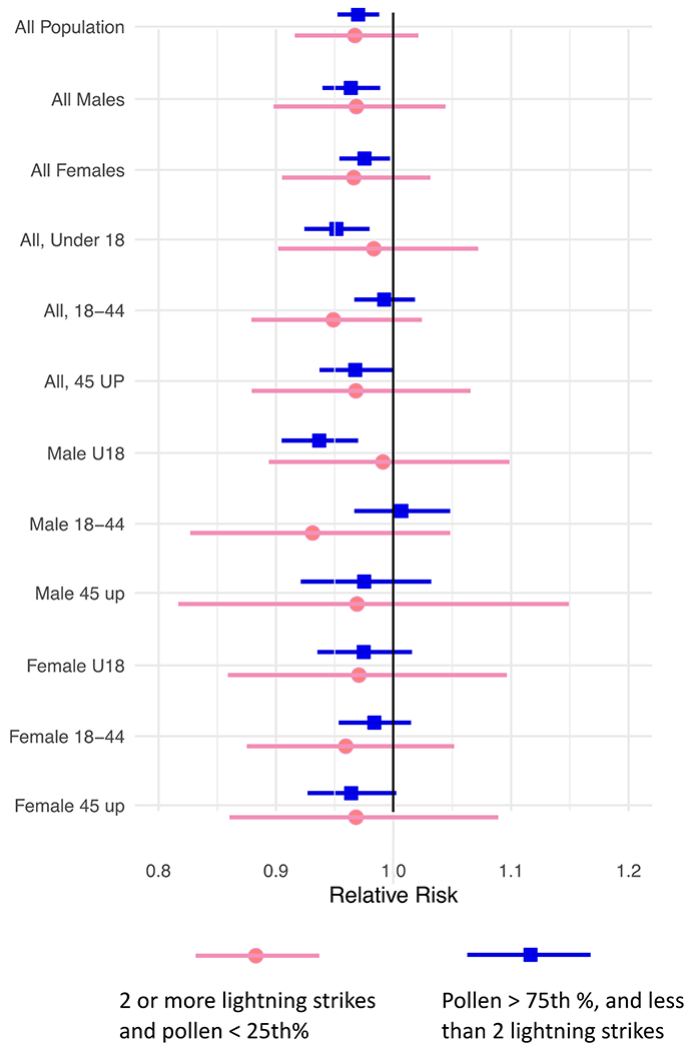
Relative risk of emergency department visits for asthma on alternative exposure days with high pollen (>= than 75 %) and low lightning (less than two strikes) and days with lightning (2 or more strikes) and low pollen (< 25th percentile).

In sensitivity analyses, we evaluated whether high pollen without thunderstorms or thunderstorms with low pollen were associated with severe asthma risk. Across all populations, no evidence of association between thunderstorms during low pollen and risk of ED visits for asthma on the day of the thunderstorm event was observed (Fig. 3). A marginally protective effect was found on high-pollen days without thunderstorms. Models were robust to temporal spline adjustment with estimates of 1.05 (95 % CI: 1.02, 1.09) using 5 knots and 1.06 (95 % CI: 1.02, 1.09) using 7 knots. Results for each age and sex subgroup with crude, crude plus weather, and fully adjusted models for 0–3 day lags along with 0–2 day lags for fully adjusted data with no missing days, and 0–2 missing days are shown in Supplemental Fig. S2.

When testing the sensitivity of our main effects model using a daily lightning and pollen interaction (as opposed to our binary thunderstorm asthma definition), we estimated the relative risk of asthma ED visits during doubly exposed days (lightning of 2+ strikes and pollen >75th percentile vs neither) to be 1.05 times (95 % CI: 1.01, 1.08) higher than a person exposed to neither with similar results at 5- and 7-degree splines (Supplemental Table S1). These results align with our model that employed the binary ‘thunderstorm asthma’ event exposure definition.

## 4. Discussion

In this study, we identified heterogeneities by sex and age in the risk of an asthma ED visit following a thunderstorm asthma event compared to days without a thunderstorm asthma event. The largest estimated subgroup effects were observed among men aged 18–44 and women >45 years, with no association observed for either males or females <18 years. This work builds upon case studies and single event descriptions of thunderstorm asthma epidemics and provides new detail into age and sex disparities that may exist for persons exposed to this unique environmental exposure. The greater risk in middle-aged males and older females is contrary to typical asthma incidence, which is most burdensome to children. The detailed age and sex exploration of this allergic-type asthma event can provide useful information for scientists and clinicians who may be unaware of the environmental risks from thunderstorm asthma events. While the overall effects seen in this study were small, the triggering extremes of storms and high pollen are expected to increase in association with a changing climate (Barnes, 2018).

When we consider the risk of asthma ED visits following a thunderstorm asthma event, we observe a different pattern from baseline asthma risk. The greatest risk of asthma ED visits following thunderstorm asthma events was observed in middle-aged males and older females – groups that have low rates for all-cause asthma ED visits. These findings support the hypothesis that thunderstorm asthma events are a unique exposure and not a simple exacerbation across all groups. Our findings of high adult risk but minimal risk in children mirror a case study from Calgary, Alberta, Canada, that found a median age of 31 years for ED visits during a thunderstorm asthma event with high bioaerosols but no change in pediatric admissions for respiratory symptoms (Wardman and MacDonald, 2002). Other research focusing on pollen exposure and independent thunderstorms as potential risk factors for respiratory outcomes (Simunovic et al., 2023; Park et al., 2022; Lappe et al., 2023) has found that these exposures are associated with higher risk of asthma. However, the unique combination of both thunderstorms and pollen that are precursors to subpollen particle formation and epidemic outcomes is still an area of sparse research.

There is little prior research into risks associated with these thunderstorm asthma events by age or sex, but the 2016 Melbourne thunderstorm asthma epidemic provides some evidence (Thien et al., 2018). In the Melbourne epidemic, 56 % of patients admitted to the ER were male (Hew et al., 2019). Our study found that the RR and absolute rates did not differ by sex. In the Melbourne event, risk of asthma ED visit or hospitalization in persons under 20 was similar on the day of the epidemic compared to control days, while adults aged 20–59 made up 57 % of asthma hospital admissions on the epidemic days compared to only 21 % of historical admissions (Thien et al., 2018). Our results, especially on the relative scale, suggest that risks associated with thunderstorm asthma events may present different patterns across the life course than overall patterns of asthma. Where usually children show highest risk of asthma ED risk, we found no evidence of higher risk of asthma ED visit following thunderstorm asthma events, but we did find some evidence of higher risk in adults.

In the 2016 epidemic event, 87 % of admissions reported a history of allergy symptoms, and 28 % reported prior asthma issues at the time of the event (Hew et al., 2019). This data suggests that outdoor exposures may be associated with higher risk of asthma outcomes during these thunderstorm asthma events (D’Amato et al., 2008). Whether these patterns of risk reflect exposure differences based on work and activities, or reflect a change in susceptibility to allergic-type asthma is unclear. A recent meta-analysis by Idrose et al (2021) found some evidence that adults with history of seasonal allergies show higher levels of eosinophils or eosinophil cationic proteins associated with asthma during high pollen seasons, which could lend credence to the eosinophil type of later asthma response (Idrose et al., 2021). In the 2016 Melbourne event, most of the cases had no documented history of asthma; however, the most severe cases did have a history of asthma, with 2/3 of these cases not using inhaled corticosteroids (Thien et al., 2018), (Cockcroft, 2018). Cockroft notes that 6 of the ten deaths were among patients with Asian or Indian ethnicity, (Cockcroft, 2018) which could be associated with work/exposure status or perhaps some underlying inflammatory response difference. Our data set was limited in having no information on race/ethnicity or prior asthma diagnosis.

Overall, the pattern of all cause asthma incidence observed in our population is consistent with existing descriptive asthma epidemiology, indicating its strength as a baseline study cohort (CDC 2017). According to the CDC, children under 18 experience higher annual asthma emergency department visits per (88.1/10,000) compared to adults 18 and up (42.1/10,000) and female patients (50.4/10,000) show higher rates compared to male patients (31.1) ((CDC) Centers for Disease Control & Prevention 2021). Emergency department visits are seen to decline across age, decreasing from 88.1 for children, 62.7 for adults ages 18–34, 36.9 for 35–64, and 18.2 for adults 65 and up ((CDC) Centers for Disease Control & Prevention 2021). In children, we see a pattern where males have a higher incidence of asthma emergency department visits (103.1) compared to females (72.5) ((CDC) Centers for Disease Control & Prevention 2021). While different studies use slightly different age groups than our study, the age-sex pattern for national severe asthma in children is consistent with our findings, with males higher in younger age groups, and male rates declining more steeply over time (QuickStats 2018; Alhanti et al., 2016; Satia et al., 2021; Miyakawa, 1991).

Our research has some limitations. Given the lack of comparison sites, the results of this study may not be generalizable beyond the Minneapolis-St. Paul area. The pollen profile of different geographic regions may influence the severity of thunderstorm asthma events, which is an important, but unstudied topic. Minnesota has only a single pollen station; however, prior research supports that pollen levels can be held constant over a region similar to our study area.^5^ Given the 20-mile radius of our study area, it is likely that our study has some exposure misclassification. However, we would expect such misclassification to bias our results towards the null (COPELAND et al., 1977; Haine et al., 2018). We additionally have limited pollution monitors, but our study area focuses on a single urban environment where available monitoring stations are more abundant. Due to data restrictions, we are unable to assess differences in risk by additional individual covariates, such as race, existing disease, or occupation, and as an ecological study, we cannot assess individual-level exposures. Other environmental covariates, such as humidity, may play an additional role in adverse respiratory outcomes and should be considered in future studies (Baldwin et al., May 2023). However, our findings provide clarity on the impacts of thunderstorm asthma across the life course by sex, particularly for males under 45, and females 18 and over. While there have been no documented epidemic thunderstorm asthma events in the United States to date, this work will hopefully add understanding and support future research given predicted patterns of changing weather and growing seasons.

## 5. Conclusions

The results of this study provide additional evidence that the phenomenon of thunderstorm asthma affects males and females differently across the life course. Where all-cause severe asthma has the highest impact on children under 19, thunderstorm asthma events are associated with absolute and relative increases of risk for males 18–44, and females 45+.

## Supplementary Material

1
