# Supplementary material for: Sex and age characteristics of thunderstorm asthma emergency department visits
Source: Hyg Environ Health Adv. Author manuscript; Available in PMC 2024 Oct 10. (PMC11466176; doi:10.1016/j.heha.2024.100099)
Supplement: 1 [file NIHMS2020517-supplement-1.docx]

Supporting Information for

Sex and Age Characteristics of Thunderstorm Asthma Emergency Department Visits

**Contents of this file**

Figures S1 to S2

Table S1

**Introduction**

This is supporting text for the manuscript including supplemental figures and tables.

Figure S1. Map of study area (in green) within the state of Minnesota, U.S.

Figure S2: Relative risk of severe asthma stratified by model type for all subgroups (All population, Male, Female, All persons age < 18, 18-44, 45 up, Male <18, Male 18-44, Male 45 up, Female <18, Female 18-44, Female 45 up) during thunderstorm asthma events at lag day 0 through lag day 3. Separate results show crude analysis adjusted for only day of week and seasonal spline with up to 3 missing days pollen estimated (red circle), full model adjusted for all covariates, with up to 3 missing days pollen estimated (blue triangle), full model adjusted for all covariates with zero days estimated (green square), and full model adjusted for all covariates with 0-2 days pollen estimated (purple cross). Lag 3 employed imputation of missing pollen values.

Table S1: Comparison of main effects, main effects with interaction terms, and relative risk of doubly exposed to pollen and storms vs. exposed to neither, for 5, 6, and 7 degrees of freedom.

| Knots per spline | Exposure | Model 1: Main effects  RR (95% CI) |  | Model 2: Interaction model RR (95% CI) | RR of exposed to both vs. neither  (95% CI) |  |
| --- | --- | --- | --- | --- | --- | --- |
| 5 | Lightning | 1.04 (1.013,1.073) |  | 1.018 (0.983,1.054) | 1.042 (1.002,1.083) | |
|  | Pollen | 0.977 (0.960, 0.995) |  | 0.971 (0.953,0.990) |  |  |
|  | Lightning*Pollen |  |  | 1.054 (1.007,1.103) |  |  |
|  |  |  |  |  |  |  |
| 6 | Lightning | 1.042 (1.013, 1.071) |  | 1.019 (0.984, 1.055) | 1.046 (1.010,1.084) |  |
|  | Pollen | 0.984 (0.814,1.189) |  | 0.978 (0.945, 1.012) |  |  |
|  | Lightning*Pollen |  |  | 1.050 (1.005,1.098) |  |  |
|  |  |  |  |  |  |  |
| 7 | Lightning | 1.035 (1.008, 1.064) |  | 1.018 (0.984, 1.052) | 1.057 (1.020,1.095) |  |
|  | Pollen | 0.997 (0.978, 1.016) |  | 0.992 (0.973, 1.012) |  |  |
|  | Lightning*Pollen |  |  | 1.047 (1.002, 1.093) |  |  |
